# Supplementary material for: ROS/RNS Balancing, Aerobic Fermentation Regulation and Cell Cycle Control – a Complex Early Trait (‘CoV-MAC-TED’) for Combating SARS-CoV-2-Induced Cell Reprogramming
Source: Front Immunol. 2021 Jul 7;12:673692. doi: 10.3389/fimmu.2021.673692 (PMC8293103; doi:10.3389/fimmu.2021.673692)
Supplement: Supplementary file 1 [file DataSheet_1.docx]

**Supplementary Figure S1**: Differences in transcript levels (normalized in RPKM values) of AOX, ADH1, LDH and Enolase in WT and clf/cwn-mutant between mock controls and between 55h auxin-treated seeds. **: highly significant (p < 0.01) differences

**

**

**

**

**

**Supplementary Figure S2**: Differences in transcript levels (normalized in RPKM) of AOX1 and AOX2 between mock control and auxin-treated seeds of *Arabidopsis thaliana* (**: highly significant (p < 0.01))
